# Material and Structural Performance Evaluations of Hwangtoh Admixtures and Recycled PET Fiber-Added Eco-Friendly Concrete for CO_2_ Emission Reduction

**DOI:** 10.3390/ma7085959

**Published:** 2014-08-19

**Authors:** Bon-Min Koo, Jang-Ho Jay Kim, Sung-Bae Kim, Sungho Mun

**Affiliations:** 1 Concrete Structural Engineering Laboratory, School of Civil and Environmental Engineering, Yonsei University, A404 Engineering Building #A, 134 Shinchon-dong, Seodaemun-gu, Seoul 120-794, Korea; E-Mail: kbm255@hanmail.net; 2 Concrete Structural Engineering Laboratory, School of Civil and Environmental Engineering, Yonsei University, A274 Engineering Building #A, 134 Shinchon-dong, Seodaemun-gu, Seoul 120-794, Korea; 3 Concrete Structural Engineering Laboratory, School of Civil and Environmental Engineering, Yonsei University, A404 Engineering Building #A, 134 Shinchon-dong, Seodaemun-gu, Seoul 120-794, Korea; E-Mail: sztk77@yonsei.ac.kr; 4 Baytech Korea, Inc., Institute Technology, Joong-il Eines Platz 9F, 513-22, Sangdaewon-dong, Jungwon-gu, Seongnam-si, Gyunggi-do 462-120, Korea; 5 Department of Civil Engineering, Seoul National University of Science and Technology, Engineering Building, 232 Gongneung-ro, Nowon-gu, Seoul 139-743, Korea; E-Mail: smun@seoultech.ac.kr

**Keywords:** eco-friendly concrete, Hwangtoh concrete, admixtures, short plastic fibers, recycling PET plastic, compressive strength, crack control, structural ductility

## Abstract

In order to reduce carbon dioxide (CO_2_) emissions and produce an eco-friendly construction material, a type of concrete that uses a minimal amount of cement, yet still retains equivalent properties to ordinary cement concrete, has been developed and studied all over the world. Hwangtoh, a type of red clay broadly deposited around the world, has traditionally been considered an eco-friendly construction material, with bonus advantages of having health and cost benefits. Presently, Hwangtoh is not commonly used as a modern construction material due to properties such as low strength and high rates of shrinkage cracking. Recent studies, however, have shown that Hwangtoh can be used as a mineral admixture to improve the strength of concrete. In addition, polyethylene terephthalate (PET) fibers recycled from PET bottle waste can be used to control shrinkage cracks in Hwangtoh concrete. Therefore, in this study, performance verification is conducted on newly developed Hwangtoh concrete mixed with short recycled PET fibers. The results show that Hwangtoh concrete has compressive strength, elastic modulus, and pH properties that are similar to these features in ordinary cement concrete. The properties of carbonation depth and creep strain of Hwangtoh concrete, however, are larger and smaller, respectively, than in ordinary cement concrete. According to flexural tests, reinforced concrete (RC) specimens cast with Hwangtoh admixtures (with and without PET fibers) possess similar or better capacities than ordinary RC specimens. The addition of PET fibers significantly improves the structural ductility of RC specimens under normal environmental conditions. However, the implementations of the concrete in aggressive environment must be carefully considered, since a previous study result indicates degradation of its durability performance in aggressive environments, such as seawater [1]. The results of this study validate the possibility of using eco-friendly Hwangtoh concrete reinforced with recycled PET fibers as a structural material for modern construction.

## 1. Introduction

Concrete is the most widely used construction material in the world due to its low cost, high availability, and simple constructability. However, the use of cement is a main contributor to high-energy usage, CO_2_ and dust emissions, natural resource depletion, air pollution, ozone layer destruction, global warming, and continuous environmental deterioration. In addition, practices related to concrete construction exploit natural resources due to a lack of careful planning, which has triggered an urgent need to find alternative construction materials to mitigate environmental damage [2,3]. In terms of health issues, there have been fervent public outcries over illnesses associated with concrete structures, such as sick building syndrome, dermatitis, allergic diseases, and atopic skin diseases. Therefore, many technologically advanced countries such as the United States and Japan are trying to reduce the use of cement and concrete while searching for alternative structural materials for housing construction [3,4,5,6,7]. Due to the high availability, consistency, reliability, and affordability of concrete, however, concrete will likely remain a primary construction material in the future. As a result of the Kyoto Protocol for regulating CO_2_ emissions and energy consumption in the international community, construction industries all over the world are trying to reduce cement usage, find alternative materials, and use recycled materials for future construction projects [4,5,6,7,8,9,10,11].

In order to develop more optimal eco-friendly concrete and reduce cement usage, research on mineral admixtures made from industrial by-products, such as fly ash, blast furnace slag, and silica fume, is being widely conducted [2,5,12,13,14]. For example, one recent alternative construction material development study explores the use of concrete mixed with crushed oyster shells for reducing coarse and fine aggregate usage [15,16]. There is a great deal of active research on the properties of concrete mixed with fly ash due to the cost advantages of fly ash over other admixtures. However, because of quality control difficulties associated with concrete mixed with fly ash, alternative studies on other types of admixture-added concrete have also been pursued. Numerous works on concrete admixtures show that the major obstacle in using admixtures in ordinary Portland cement (OPC) concrete for actual construction is the 30% maximum limit in the cement substitution ratio. According to most studies, when the maximum substitution percentage is violated, reductions in properties of strength, crack resistance, and overall material performance are observed [14,17,18,19,20]. Therefore, a solution to the problem of how to increase the admixture substitution ratio in a concrete mix without decreasing the final performance of the material is needed in order to develop useable eco-friendly concrete. In addition, a simple method of recycling industrial waste products for admixture applications in concrete mixtures must be established in order to develop innovative eco-friendly concrete that is not harmful to human health.

Hwangtoh (*i.e.*, a type of red clay) is receiving substantial interest as a possible eco-friendly construction material. Accordingly, Hwangtoh is the subject of much industry research [4,21,22,23,24]. Hwangtoh is a Kaolin material with abundant reserves all over the world, and has traditionally been considered an eco-friendly material. According to recent research, Hwangtoh can be used as a mineral admixture for eco-friendly concrete due to its pozzolanic characteristics [4,22,25,26]. However, any Hwangtoh admixture replacement ratio greater than 15%–20% of the total cement weight sharply degrades the material properties of Hwangtoh concrete. Therefore, a Hwangtoh admixture replacement ratio of 15%–20% of the total cement weight in a concrete mix has generally been accepted as the maximum replacement ratio [27,28]. Recent experimentation with Hwangtoh powder blended with mineral admixtures has been pursued in order to improve the pozzolanic properties of Hwangtoh, and to increase its replacement ratio in a concrete mix [28,29]. The findings of these studies show that blending Hwangtoh with slag powder cement provides an optimal proportion ratio to maximize the strength capacity of Hwangtoh concrete.

In order to use Hwangtoh as a structural material, the inherent shrinkage-cracking problem associated with Hwangtoh must be resolved. Based on the findings of research on fiber reinforced concrete and its ability to control cracking in the cement matrix, studies pertaining to the application of short plastic fibers in Hwangtoh concrete have been conducted [9,30,31,32,33,34]. In conjunction with efforts by the plastic fiber industry to recycle plastic waste so that waste materials can be utilized for manufacturing secondary products, several studies have been carried out in recent years to optimize-recycling of plastic waste into plastic fibers for making eco-friendly concrete [1,10,35,36,37,38,39].

Previous research on Hwangtoh has mostly consisted of studies that investigate the basic material and mechanical properties of Hwangtoh, together with mixing proportions and procedures, to evaluate its constructability [4,21,22,23,24,27,28,29]. The aim of this study is to develop a type of Hwangtoh concrete with sufficient compressive strength to meet the standards of modern construction by adding Hwangtoh-slag admixtures, and to examine the shrinkage crack-controlling capability of Hwangtoh concrete when short recycled PET fibers are added to the mix. With the goal of developing an alternative construction material with healthy and eco-friendly qualities, the focus of this work is evaluation of the newly developed Hwangtoh concrete material in terms of its durability and structural performance capacities. The material and durability properties (*i.e*., shrinkage strain, compressive strength, elastic modulus, pH, carbonation depth, and creep strain) of plain Hwangtoh concrete, as well as the structural properties (*i.e.*, load-deflection relationships, ultimate failure load, ductility index, and cracking patterns) of RC beams cast with Hwangtoh concrete and recycled PET fibers are examined.

## 2. Material Properties and Mix Proportions

### 2.1. Hwangtoh

Hwangtoh has traditionally been considered an eco-friendly construction material for use in plastering walls and ondol (traditional Korean underfloor heating systems) in Korea. The main advantages of Hwangtoh are its high heat absorption capacity, auto-purification properties, antibiotic properties, and infrared ray emission capability [23]. However, the development of Hwangtoh has not been actively pursued in modern constructions, and the material has not been utilized in modern buildings due to its low compressive strength and low dry shrinkage crack resistant capacity. According to the results of recent research, however, Hwangtoh can be used as a substitute material for natural pozzolanic admixtures, such as flyash or pozzolan [4,22,24]. The potential of Hwangtoh as a construction material, especially in the construction of housing, is extremely promising because its chemical and mineralogical properties are similar to those of Metakaolin and Kaolinite. Silicon dioxide (SiO_2_) forms stable pozzolan products in Hwangtoh by combining with cement hydrate Ca(OH)_2_ at room temperature, thus acting as a pozzolan material, such as fly ash or silica fume [40]. The chemical equation for the pozzolanic reaction in Hwangtoh is written as follows:


3Ca(OH)_2_ + 2SiO_2_ → 3CaO·2SiO_2_·3H_2_O (1)

### 2.2. Recycled PET Fibers

Polyethylene terephthalate (PET) is the most widely used plastic material in the world for manufacturing secondary products such as beverage bottles. However, PET bottles are commonly thrown away after a single use, which contributes to significant environmental problems. Therefore, methods to recycle PET bottle waste have been examined in order to improve PET recycling applications. The use of PET plastic fibers recycled from PET bottle waste in construction projects has been actively studied. Short PET fibers can be utilized to effectively reduce shrinkage strain and to enhance the ductility of concrete, especially to mitigate the inherent shrinkage problem of Hwangtoh concrete [35,36,41,42].

### 2.3. Optimal Hwangtoh Replacement Ratio Selection

Our experiments aimed to reduce the use of ordinary Portland cement (OPC) in concrete mixes as much as possible by replacing OPC with Hwangtoh and blast furnace slag powders. Before the actual casting of the specimens, trial tests were performed to find the optimal replacement ratio of Hwangtoh and blast furnace slag powders. In a previous study on the use of Hwangtoh powder as an admixture in a concrete mix, serious degradations in concrete performance were observed when the Hwangtoh to cement substitution ratio exceeded 20%. Therefore, a substitution ratio exceeding 20% is generally not recommended in concrete mix proportions [27,28]. Significant workability degradation has also been reported in many Hwangtoh concrete studies [4,21,27,43]. Hence, it is important to develop a concrete mix proportion using admixtures of Hwangtoh and blast furnace slag powder without decreasing the overall properties of strength, crack resistance, and workability in the final material. While a reduction in workability can be controlled by adding a water-absorbing chemical admixture to satisfy the target slump, this study adheres to the principle of adding the maximum amount of blast furnace slag, Hwangtoh, and other eco-friendly admixtures in the mix proportion.

**Figure 1:**
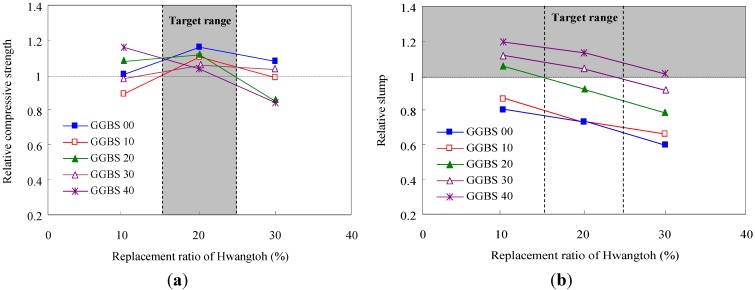
Compressive strength and slump of Hwangtoh concrete according to pre-tests (**a**) Compressive strength and (**b**) Slump.

Trial mix proportion tests were carried out by setting the water-to-cement ratio (W/C) as a constant, while varying the Hwangtoh and slag powder replacement ratios. The results from the trial tests are shown in Figure 1. The trial test results reveal that when the Hwangtoh replacement ratio was 20%, little variation in strength (independent of the slag replacement ratio) was observed. The workability decreased as the Hwangtoh replacement ratio increased due to the high porosity of Hwangtoh, which causes rapid water absorption during the mixing process. However, as shown in Figure 1b, when OPC, Hwangtoh, and blast furnace slag powders were mixed, the slump of the mix was higher than the slump of the OPC mix. This result is likely caused by the relatively smooth surface texture of blast furnace slag powder particles, which reduces both water molecule attachments on particle surfaces and friction in the cement paste-aggregate interface.

In this study, replacement ratios of 20% and 30% were used for the Hwangtoh and slag powders, respectively, for a 50% total cement replacement ratio in the concrete mix. In order to improve the high rate of shrinkage in Hwangtoh concrete, a 0.5% volume of short plastic fibers made from recycled PET plastic bottles was also added to the mix. From the trial tests, it was found that the optimal PET fiber volume percentage to control crack formation in the concrete was approximately 0.5%.

The material properties of the Hwangtoh used in this study are shown in Table 1. Two types of Hwangtoh were employed in the study, namely K-type and C-type Hwangtoh produced in Korea and China, respectively. Recycled PET fiber used in the study was an embossed type with dimensions of 0.2 mm × 1.3 mm × 50 mm and density of 1.38 g/cm^3^. Elastic modulus, tensile strength, and ultimate elongation percentage of the fiber were 1.2 × 10^4^ MPa, 420.7 MPa, and 11.2%, respectively. The final mixture proportions selected for the experiments are shown in Table 2.

**Table 1:** Chemical composition of Hwangtoh.

Composition type	SiO_2_	Al_2_O_3_	Fe_2_O_3_	MgO	CaO	K_2_O	TiO_2_	Na_2_O
KH	42.5	36.6	4.05	0.69	0.57	0.41	0.23	0.18
CH	57.0	18.0	6.15	0.32	8.91	0.2	0.25	0.20
GGBS (Slag)	41.2	34.2	11.7	8.81	-	0.31	-	0.29

**Table 2:** Concrete mixture proportions.

Specimens	W/B (%)	S/A (%)	Replacement ratio of Hwangtoh (%)	Replacement ratio of GGBS (%)	Unit weight (kg/m^3^)						RPET fiber (%)	SP (%)
					W	C	HT	GGBS	S	G		
Control	55	44.8	-	-	186.7	339.4	-	-	746.7	958.1	-	0.7
SC			-	30		237.6	-	101.8			-	
KH			20	-		271.5	67.9	-			-	
CH			20	-		271.5	67.9	-			-	
KHS			20	30		169.7	67.9	101.8			-	
CHS			20	30		169.7	67.9	101.8			-	
KHSP			20	30		169.7	67.9	101.8			0.5	
CHSP			20	30		169.7	67.9	101.8			0.5	
RPET			-	-		339.4	-	-			0.5	

## 3. Experiment Methods

### 3.1. Compressive Strength and Elastic Modulus Tests

**Figure 2:**
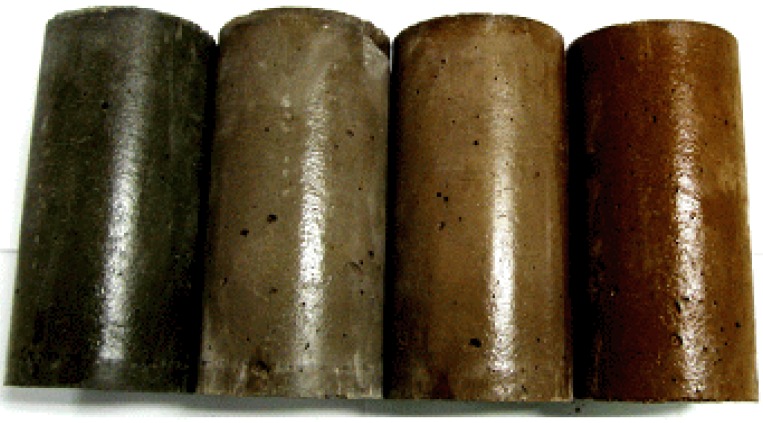
100 mm × 200 mm Hwangtoh and slag cement concrete cylindrical specimens.

A total of 27 specimens were used for compression and elastic modulus tests. Hwangtoh concrete cylinders with dimensions of 100 mm × 200 mm were compression tested at 28 days from initial casting. These cylindrical specimens were tested with a universal testing machine (UTM) with a maximum load capacity of 2000 kN. The compressive strength of each specimen type was determined by averaging three specimen strength values. After casting, the cylindrical specimens were covered with plastic wrap for 24 h, after which the forms were removed. The specimens were then stored in a humidity chamber at a temperature of 20 ± 1 °C and a humidity of 60% ± 5% until testing. Compressive strength and elastic modulus experiments were performed according to Korean Standards 2405 and 2438, respectively [44,45]. A photo of the cylindrical specimens mixed with Hwangtoh and slag cement is shown in Figure 2.

### 3.2. Method for pH Tests

In order to measure the pH of Hwangtoh cement paste, Hwangtoh specimens with W/C of 55% were prepared. For comparative purposes, additional specimens made exclusively from Korean and Chinese Hwangtoh (called KH100 and CH100, respectively) were prepared. The formwork was removed after 24 hours of casting. In order to prevent evaporation of moisture from the specimens, the specimens were wrapped with plastic wrap before being put into a curing chamber with a temperature of 20 ± 2 °C and a relative humidity of 64% for 200 days. The loss of alkali residuals in the powder (sieve size 300 μm) was minimized by drying the power in a drying chamber for 24 h prior to testing. A volume of 3.0 g of the dried power was subsequently mixed with 40 mL of distilled water. After stirring the wet powder for 10 min, the pH was measured using a pH-measuring device. The pH correction factor was used to set the initial pH values at 4.01, 7.00, and 10.01 prior to measurement [46,47].

### 3.3. Carbonation Depth Test

**Figure 3:**
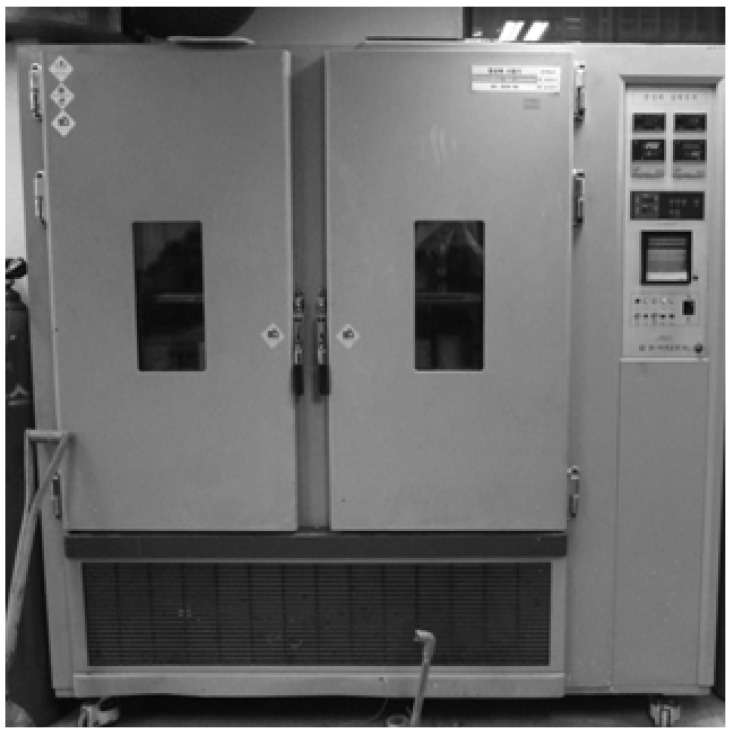
Carbonation test chamber.

In order to evaluate the effect of Hwangtoh on the carbonation properties of Hwangtoh concrete, carbonation depths at various curing ages were measured. The accelerated carbonation test was performed following the test guidelines of KS F 2584 [48]. The specimens were wet-cured for four weeks, followed by dry curing for another four weeks. Before the test, the specimens were coated with epoxy at both ends. The accelerated carbonation test was performed in a carbonation test chamber shown in Figure 3 under temperature, relative humidity, and CO_2_ conditions of 20 ± 2 °C, 60% ± 5%, and 5% ± 0.2%, respectively. Carbon depth was measured based on the location of phenolphthalein color change at testing times of one, four, eight, 12, and 25 weeks, following the guidelines of KS F 2596 [49].

### 3.4. Creep Test Method

**Figure 4:**
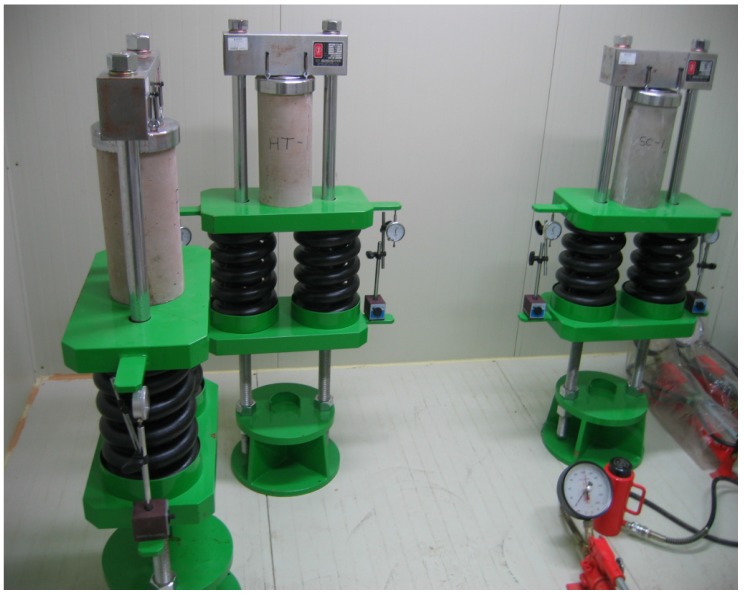
Creep test setup.

Currently, there are no guidelines available for the creep testing of recycled PET fiber-added Hwangtoh concrete. Accordingly, the concrete creep test guidelines of ASTM C 512 were used for this study [50]. A cylindrical specimen with a diameter of 150 mm and a height of 300 mm, containing air cured for seven days, was used for the test herein. The creep test was performed inside a humidity chamber with a temperature and relative humidity of 22 °C and 60%, respectively. The specimens of slag cement concrete, Hwangtoh concrete, and recycled PET fiber-added Hwangtoh concrete were creep tested by applying a load equivalent to 40% of the load used for the 28-day compressive strength tests, using a creep test device, as shown Figure 4. The creep load was applied to the specimens by compressing the springs attached to the bottoms of the specimens using an actuator. When the target load was applied, the spring length was fixed by a bolt that secured the steel plate to the bottom of the specimen. After application of the load, dial gauges were installed on the left and right sides of the specimen to measure deflection at an interval of seven days.

### 3.5. Drying Shrinkage Cracking Test Methods

**Figure 5:**
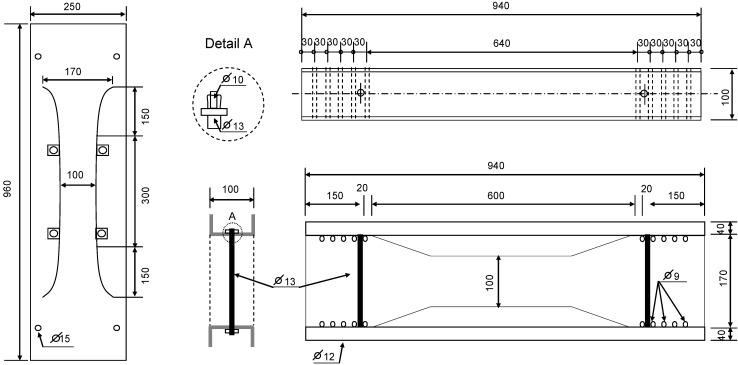
Restrained frame and concrete mold dimensions and details (units: mm).

To evaluate the drying shrinkage cracking capacity of Hwangtoh concrete, free and restrained drying shrinkage strain experiments were performed according to Korean Standard 2595, which details the drying shrinkage cracking test method for restrained concrete [51]. The specimens were cast and placed in a humidity chamber at a curing temperature of 20 ± 1 °C and a relative humidity of 60% ± 5% until testing. Removal of the form was performed seven days after casting. In order to measure the restrained drying shrinkage strain after removal of the form, strain gauges were attached to the central part of the upper and lower surfaces of the specimen and to the central part of the restraining plate in order to measure the concrete and restraining plate strains, respectively. The specimens were checked daily and the location and time of cracking were recorded. The dimensions and shape of a drying shrinkage cracking specimen are shown in Figure 5.

### 3.6. Reinforced Concrete (RC) Flexural Test Methods

**Figure 6:**
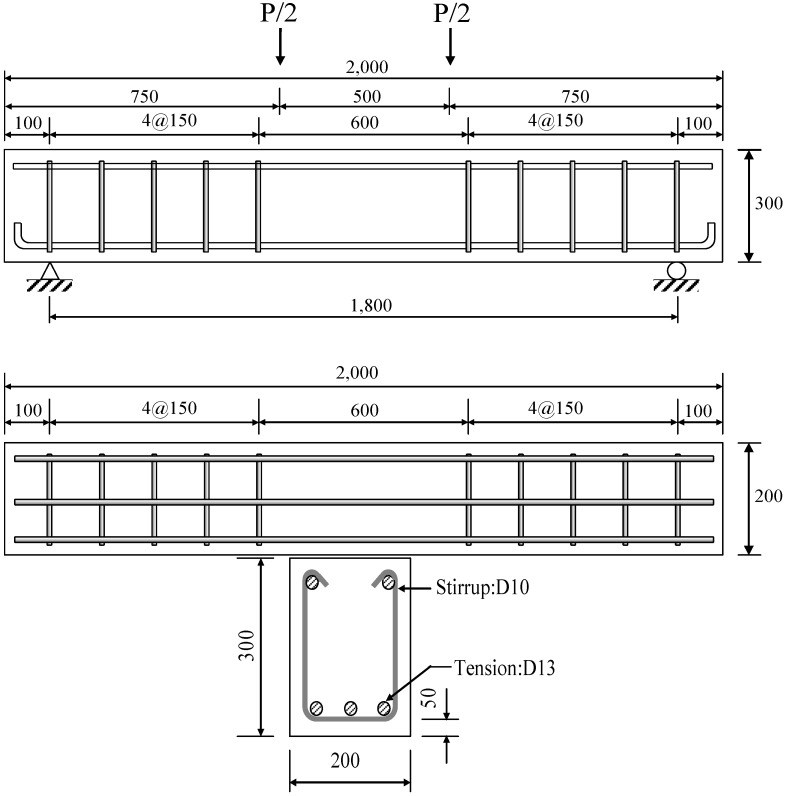
Dimensions and details of RC beams (units: mm).

**Figure 7:**
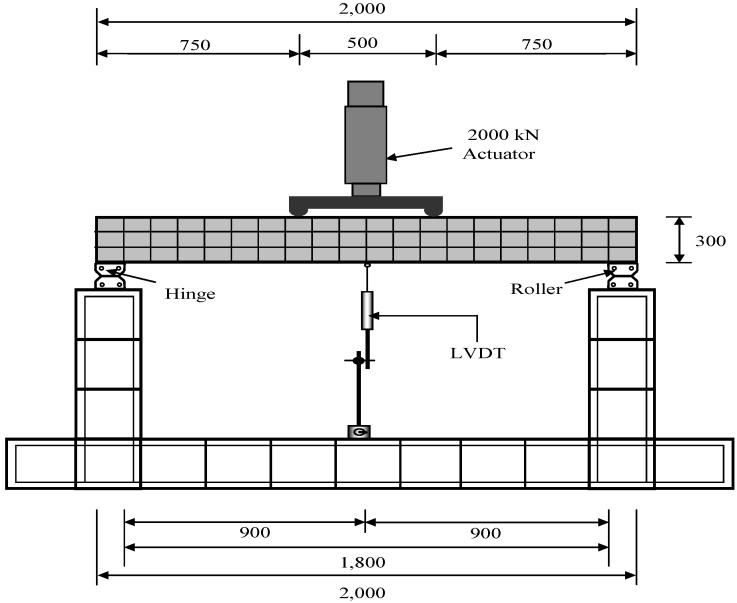
Test setup and data acquisition locations (units: mm).

All specimens were tested for flexural strength 28 days after casting. The specimens were reinforced with three D13 rebars (with a diameter of 13 mm) as tension reinforcement and D10 rebars (with a diameter of 10 mm) with 150 mm spacing as shear reinforcement. The dimensions and details of an RC flexural test specimen are shown in Figure 6. The specimens were tested using a four-point loading setup with hinge-roller supports, as shown in Figure 7. Stroke control loading with a vertical displacement rate at the mid-span of 0.025 mm/sec was applied with a UTM with a capacity of 2000 kN. A strain gauge and a linear variable differential transducer (LVDT) were attached to the bottom of the concrete surface of the specimen at the mid-span point, in order to measure the tensile strain and vertical deflection, respectively. Crack initiation and propagation were visually monitored and recorded throughout the flexural test.

## 4. Experiment Results and Discussion

### 4.1. Compressive Strength and Elastic Modulus

**Figure 8:**
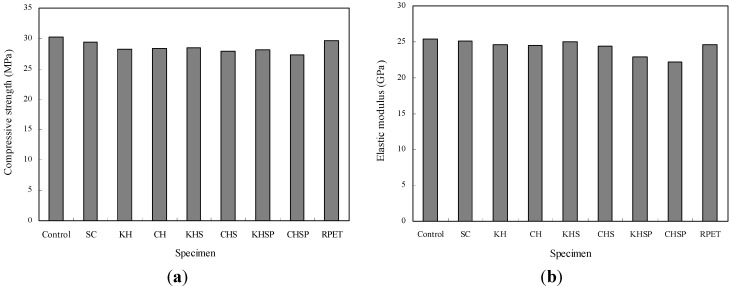
Compressive strength and elastic modulus of Hwangtoh concrete from main tests (**a**) Compressive strength and (**b**) Elastic modulus.

The compressive strength test results for the Hwangtoh concrete are shown in Figure 8a. Control and slag concrete (SC) specimens were cast with plain OPC and slag cement concrete, respectively. To define our working acronyms, KH and CH specimens were cast with concrete containing Korean and Chinese Hwangtoh powder, respectively, while KHS and CHS specimens were cast with Korean and Chinese Hwangtoh with slag powder concrete, respectively. KHSP and CHSP specimens were cast with KHS and CHS concrete containing PET fibers, respectively, and an RPET specimen was cast with plain concrete containing PET fibers. As shown in Figure 8a, the control specimen demonstrated the highest compressive strength of 30 MPa. In contrast, an SC specimen, the KH and CH specimens mixed with Hwangtoh, and the KHS and CHS specimens mixed with Hwangtoh and slag exhibited lower capacities of compressive strength. These results demonstrate that the pozzolanic reactions of Hwangtoh and slag powders are not as active as the pozzolanic reaction of cement. This in turn causes less hydration in the mix, and produces an internal structure that is less dense. Recycled PET fiber-reinforced KHSP and CHSP specimens exhibited the lowest compressive strength out of all the specimens. In particular, the CHSP specimen displayed the lowest compressive strength (27 MPa) due to the addition of PET fibers. The behavior of the fibers within the matrix can be explained by drawing an analogy between fibers and internal voids, which decrease the effective cross-section and strength of a specimen. However, a compressive strength comparison between the different Hwangtoh concrete specimens and the control specimen show that the strength reduction is less than 2%–6% and is thus relatively insignificant for structural member construction.

The elastic modulus test results for the various Hwangtoh concrete specimens are shown in Figure 8b as a bar graph. Similar to the compressive strength test results, the control specimen exhibited the highest elastic modulus at 25 GPa, while the moduli of the other Hwangtoh concrete specimens were in the range of 23–25 GPa. The results of the compressive strength and elastic modulus test for the Hwangtoh concrete and plain concrete specimens reveal similar capacities among the types of material. Indeed, the differences between the two materials are within an acceptable margin, indicating that their capacities may be considered equivalent for structural usage.

### 4.2. Compression Failure Behavior

**Figure 9:**
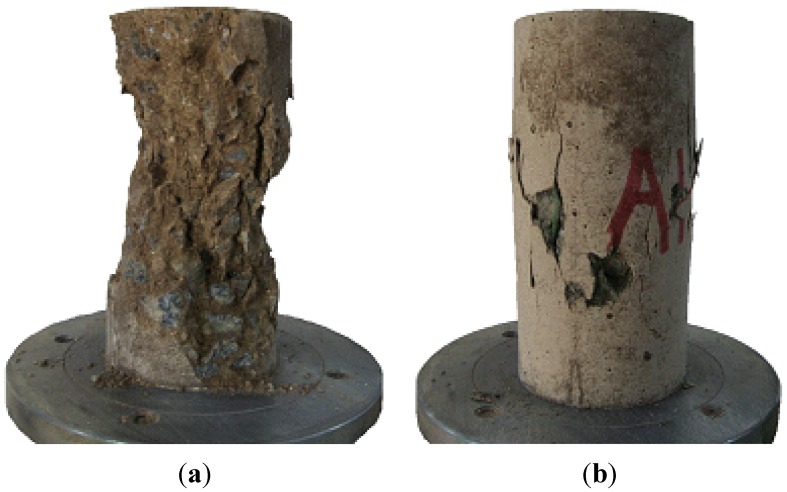
Failure mode of cylindrical Hwangtoh concrete specimens (**a**) Hwangtoh specimen without fibers and (**b**) Hwangtoh specimen with RPET fibers.

The compressive failure modes of the cylindrical specimens are shown in Figure 9. As illustrated in Figure 9a, the failure mode of the Hwangtoh concrete was in line with the cone failure type, which is a typical concrete failure mode observed in compressed specimens. However, the recycled PET fiber-reinforced Hwangtoh concrete specimens failed due to a large expansion strain in the center, along with multiple vertical cracks, as shown in Figure 9b. These failure modes indicate that the recycled PET fibers restrained concrete spalling and crack growth. The fibers also dispersed internal stresses uniformly over the cross-section, thereby preventing localized failure. These results suggest that synthetic fibers, such as recycled PET and polypropylene (PP) fibers, are beneficial in preventing premature concrete failure. In addition, recycled PET fiber is effective in controlling crack growth in Hwangtoh concrete.

### 4.3. Measurement of pH

**Figure 10:**
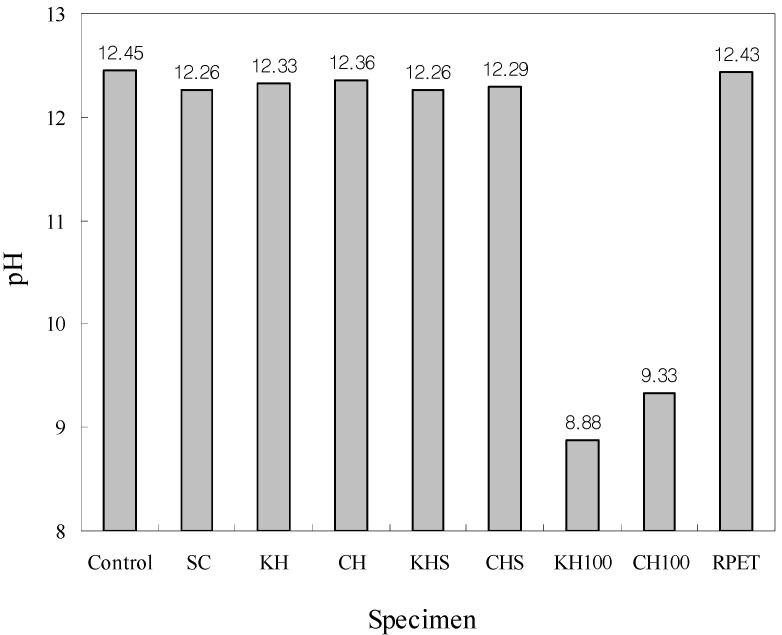
Results of pH measurement.

Measurement data on pH values are shown in Figure 10. The figure shows that the pH of the control specimen (general OPC concrete) was approximately 12.45, while most of the other specimens (with the exception of the PET fiber-reinforced Hwangtoh specimen) had lower pH values. The results show that, as the amount of slag and Hwangtoh admixtures increased (thereby decreasing the amount of OPC in the mix), the pH values decreased. In addition, the results show that PET fibers do not have a significant effect on the pH of concrete. The measured pH values of KH 100 and CH 100 specimens were approximately 8.88 and 9.33, respectively, showing a reduced pH, which is characteristic of Hwangtoh. The results of a previously published study report that the pH of calcium oxide (Ca(OH)_2_) is approximately 12.5 [52]. Thus, the pH value of the control specimen was controlled by calcium oxide, as expected. The pH values of the Hwangtoh specimens, however, were lower due to reduction in the amount of calcium oxide production caused by the partial replacement of OPC with slag and Hwangtoh in the Hwangtoh specimens. It is important to note that the amount of calcium oxide production during the hydration processes of slag and Hwangtoh concrete is significantly less than the amount of calcium oxide production in OPC, thereby resulting in lower pH values in slag and Hwangtoh concrete.

### 4.4. Carbonation Depth

**Figure 11:**
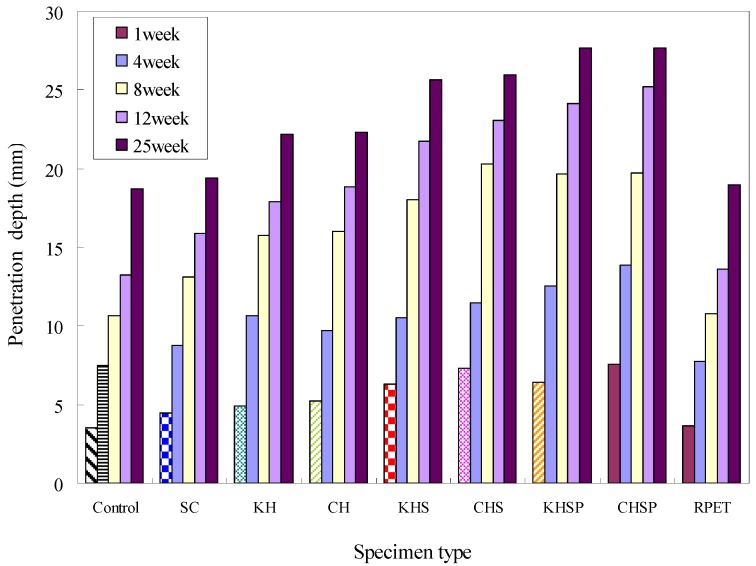
Carbonation depth results.

Carbonation depth data are shown in Figure 11. The measurements were obtained at one, four, eight, 12, and 25 weeks from the start of the test. The results show that the control and PET fiber-added concrete specimens had the least carbonation depth. Carbonation depth values ascend in order from slag cement, Hwangtoh, Hwangtoh slag cement, and Hwangtoh slag cement-PET fiber specimens, with slag concrete having the lowest carbonation depth. The basic cause of carbonation in concrete is penetration of CO_2_ gas into the concrete. In this way, the interior microstructure compactness of concrete dictates its carbonation resistance. In addition, interior defects or interfaces decrease the carbonation resistance of concrete. Based on these fundamental mechanics, it is logical that slag and Hwangtoh-added concrete would have less carbonation resistance, because lesser amount of calcium oxide are produced in these specimens than in OPC specimens. Additionally, the addition of PET fibers increases the damage-like interfaces in the engineered specimens, resulting in lower carbonation resistance.

### 4.5. Creep Strain

**Figure 12:**
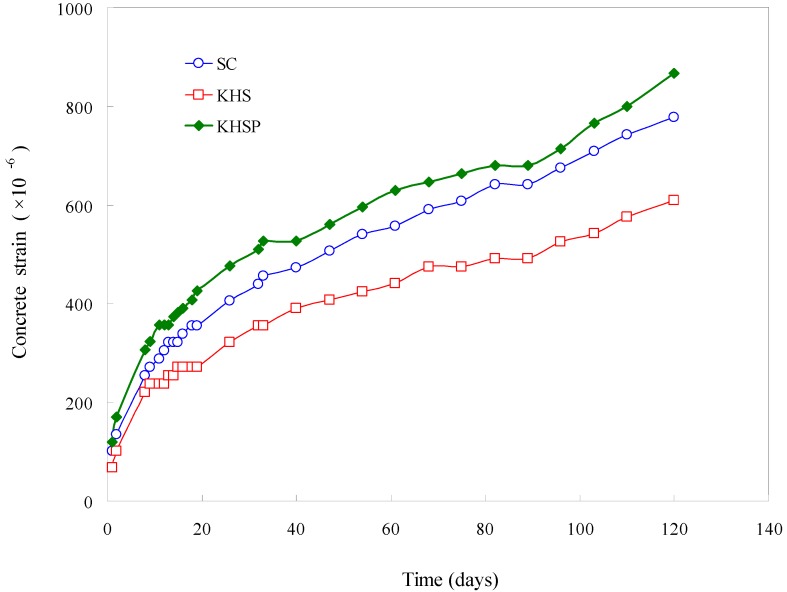
Creep strain test results.

The creep strain test results for slag concrete, Hwangtoh concrete, and Hwangtoh-slag-PET fiber concrete measured up to 120 days are shown in Figure 12. The strain drastically increased in the early testing period within the initial 20 days, and then the rate of strain increase became less drastic. The creep strain measurements at 120 days ascend in order from Hwangtoh-slag (610 × 10^−6^), slag (780 × 10^−6^), to Hwangtoh-slag-PET fiber (871 × 10^−6^) concrete, with Hwangtoh-slag concrete having the lowest creep strain at 120 days. The creep strain test results demonstrate that Hwangtoh is less susceptible to creep strain than slag cement, because the proportion of cement is reduced by the addition of the Hwangtoh admixture in the Hwangtoh-slag concrete mix relative to the slag concrete mix. In addition, the addition of recycled PET fibers increased the interfaces in the concrete, resulting in significant creep strain from the closing of interfaces as the duration of loading increased.

### 4.6. Free and Restrained Drying Shrinkage Cracking Behaviors

**Figure 13:**
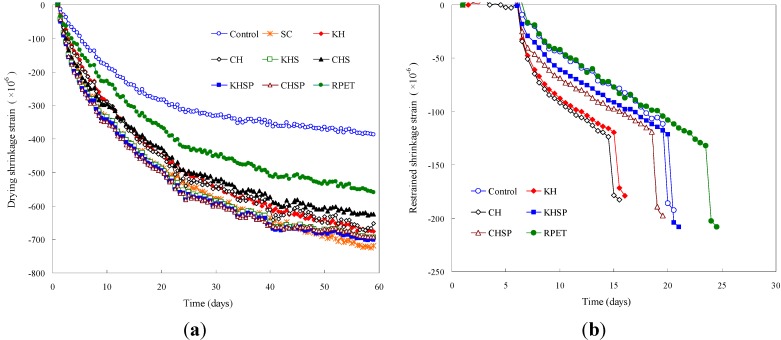
Free and restrained drying shrinkage strain test results (**a**) Free drying shrinkage strain results and (**b**) Restrained drying shrinkage strain and cracking results.

The measured free drying shrinkage strain in the test specimens is shown in Figure 13a. The control specimens exhibited the lowest free drying shrinkage strain, while the PET fiber-reinforced Hwangtoh concrete specimens showed 150%–200% more shrinkage strain than the control specimen at an age of 59 days. This result is indirectly explained by the results of the compressive strength test for the Hwangtoh concrete reinforced with recycled PET fibers. The reduced compressive strength of concrete reinforced with Hwangtoh admixture and recycled PET fibers is due to the lower density of its internal matrix microstructure and the increased amount of voids from fiber interfaces, which cause the material to be more susceptible to strain and cracking. The increased free drying shrinkage strain of the Hwangtoh concrete is the direct consequence of the high porosity and water absorption characteristics of the material due to the addition of the Hwangtoh admixture [24]. The introduction of recycled PET fibers into the cement matrix increases the pore volume and reduces the rigidity, which also increases the strain [35,53].

For the restrained drying shrinkage strain tests, the cracking times from the initial casting for all specimens are listed in Table 3. As measured, the curves of the restrained drying shrinkage strain as a function of curing time are shown in Figure 13b. The earliest full-through crack occurrence times were observed for the CH and KH specimens at 15 and 16 days, respectively, while the latest occurrence of cracking was observed for the PET fiber-reinforced RPET specimen at 25 days. The control specimen had full-through cracks at 20 days, and the CHSP and KHSP specimens had a full-through crack at 19 and 21 days, respectively. As observed from the test results, the full-through cracking time of the Hwangtoh concrete specimen was about five days earlier than the full-through cracking time of the control specimen. The CHSP and KHSP specimens exhibited times that were four to five days later than the time of the CH and KH specimens due to the crack-controlling characteristics of the PET fibers. Indeed, the delay in full-through crack formation in PET fiber-reinforced material comes from the control of shrinkage micro-cracks due to the bridging effects of PET fibers in the matrix.

**Table 3:** Restrained drying shrinkage cracking test results.

Specimens	Full-through cracking time (Days)
Control	20
KH	16
CH	15
KHSP	21
CHSP	19
RPET	24

### 4.7. Load-Deflection Relation

**Figure 14:**
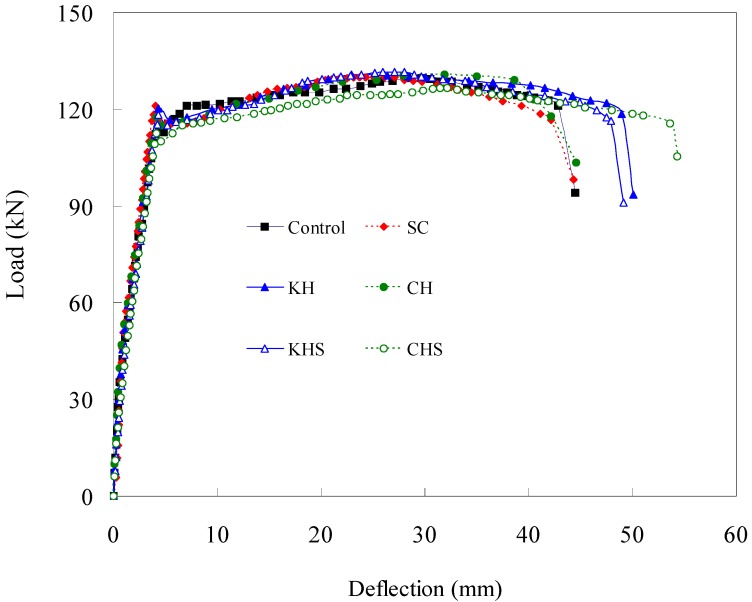
Load-deflection curves for Hwangtoh and slag cement concrete specimens.

**Figure 15:**
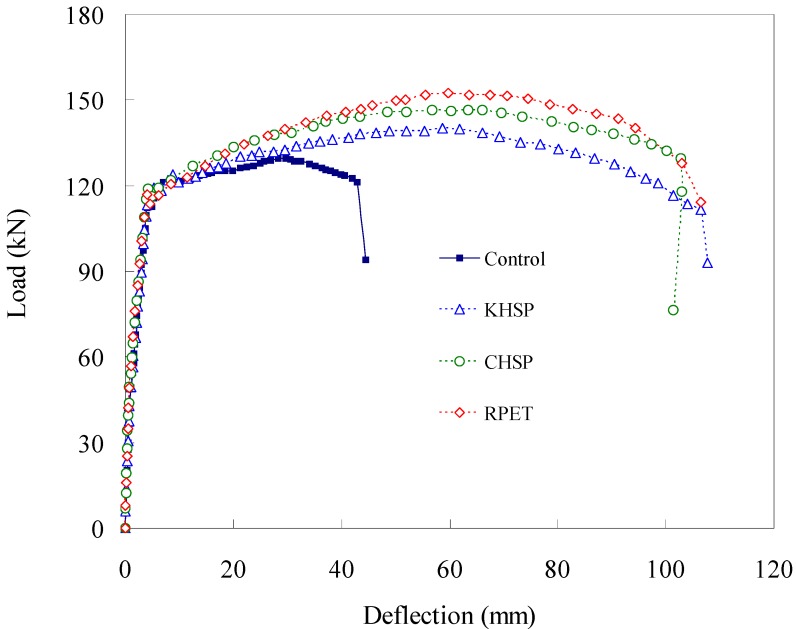
Load-deflection curves for recycled PET fiber RC beams.

The load-deflection test results for different admixture-added RC specimens are shown in Figure 14 and summarized in Table 4. As seen in Figure 14, all of the specimens with different admixtures exhibited similar elastic behaviors prior to the occurrence of until cracking. Before the yielding of the tension rebars, crack initiation occurred earlier in the control specimen than in the Hwangtoh concrete specimens. However, once the rebars yielded, the Hwangtoh concrete and the control specimens showed similar overall behavior with slight deviations depending on the admixture type. The flexural capacities of the specimens reinforced with recycled PET fibers are shown in Figure 15. The recycled PET fiber-reinforced specimens exhibited better flexural capacity than the control specimen. From the results, it is safe to assume that recycled PET fibers can control shrinkage cracks and improve the structural ductility of reinforced Hwangtoh concrete members. However, the CHSP and KHSP specimens showed a lower flexural capacity than the RPET specimen. This behavior can be attributed to the lower bond strength between Hwangtoh concrete and recycled PET fibers. Accordingly, this issue needs to be addressed in future studies on the bond capacity of Hwangtoh concrete.

**Table 4:** Flexural strength test results for Hwangtoh concrete beams.

Specimens	P_cr_ (kN)	∆_cr_ (mm)	P_y_ (kN)	∆_y_ (mm)	P_u_ (kN)	∆_u_ (mm)	Ductility Index(∆_u_/∆_y_)	Relative Ductility Index
Control	42.5	0.84	112.1	4.09	129.56	29.6	7.24	1
SC	50.8	0.94	121.1	4.03	129.95	26.1	6.48	0.90
KH	45.5	0.82	120.2	4.29	130.54	26.4	6.15	0.85
CH	46.8	0.77	117.6	4.16	129.16	38.6	9.28	1.28
KHS	43.9	1.02	118.2	4.35	131.52	27.1	6.23	0.86
CHS	45.3	1.18	112.5	4.17	126.62	31.5	7.55	1.04
KHSP	42.9	0.76	117.2	4.29	140.00	59.8	13.94	1.93
CHSP	49.4	0.79	118.8	4.27	146.60	63.4	14.85	2.05
RPET	49.0	0.74	117.0	4.11	152.39	59.8	14.55	2.01

Notes: *P* and *∆* are the load and deflection of the member, respectively; *cr* is the initial crack condition; *y* and *u* are the yield and ultimate conditions, respectively.

### 4.8. Ductility Index

The ductility index is used to evaluate the energy absorbing capacity of reinforced concrete members, which, along with the strength, is one of the most important factors in evaluating the safety of a structural member. In this study, the ductility index (Δu/Δy), which is defined as the ratio of deflections at the ultimate load, Δu, to the deflections at the time of tension rebar yielding, Δy, is used to evaluate the safety of a member (see Table 4). As calculated, the results show that the specimens with and without recycled PET fibers had similar ductility indices, even though slight deviations were observed. The load-deflection relationships shown in Figure 14 and Figure 15 clearly validate the possibility of using Hwangtoh concrete as an alternative material to standard concrete.

The recycled PET fiber reinforced specimens exhibited better ductility indices than the non-reinforced specimens. As shown in Table 4, the recycled PET fiber-reinforced concrete specimens had relative ductility indices in the range of 1.93–2.05, which were approximately two fold greater than the ductility indices of the specimens without recycled PET fibers. The higher ductility index of PET fiber-reinforced concrete is due to the crack-bridging and controlling effects of recycled PET fibers in the concrete matrix. In addition, the fibers are beneficial in transferring tensile stresses in the member by delaying the fiber pull-out time and inhibiting crack propagation. The control of crack growth during loading slows the overall flexural failure of an RC specimen, thereby improving its strength and ductility (*i.e.*, the energy absorbing capacity). These results validate the results of a previous study by Lin *et al.* [54], wherein fiber-reinforced concrete is shown to be superior in resisting tensile stresses due to its crack-bridging capability and fiber pull-out resistance.

### 4.9. Cracking Modes

**Figure 16:**
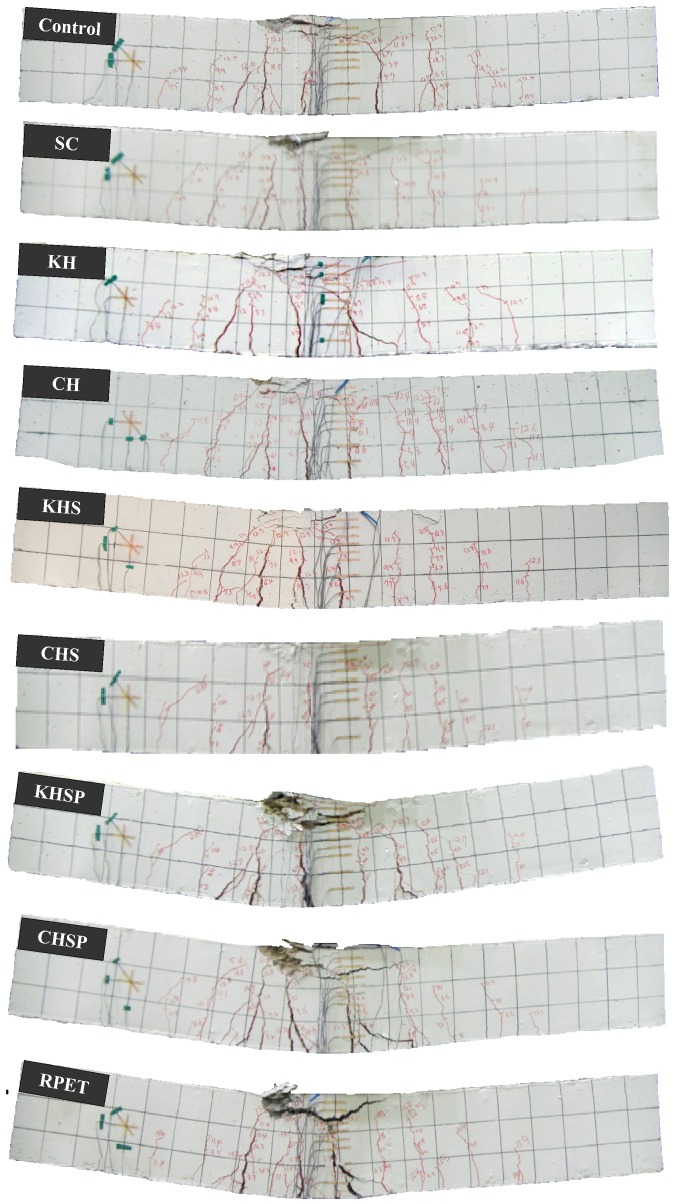
Failure mode and crack patterns of various RC specimens.

The failure modes of the RC specimens are shown in Figure 16. In order to understand the failure behavior of specimens mixed with a Hwangtoh admixture, the stress distribution and the crack growth for each load step were recorded until failure. All specimens were designed with a span-to-depth ratio (A/D) of 2.6, and were reinforced with shear stirrups to enforce flexure failure. The control specimen exhibited typical flexural tensile cracking failure, simultaneous to yielding of tension rebar. When the ultimate failure load was reached, catastrophic flexural failure occurred. All the specimens with the Hwangtoh admixture demonstrated failure behavior similar to the failure behavior that of the control specimen. The AH and AHS specimens mixed with Korean Hwangtoh powder, however, exhibited a slightly higher flexural capacity than the control specimen. The KHSP, CHSP, and RPET specimens showed partial crushing failures in the upper compressive region followed by rebar yielding. In contrast to the typical rebar yielding flexural failure behavior observed in the specimens without recycled PET fibers, compressive crushing failure behavior was observed in the specimens with PET fibers. This observation is due to the improved tensile stress transferring and crack-controlling capabilities of the PET fibers in the bottom tensile region of the PET fiber-reinforced concrete. In addition, the Hwangtoh concrete specimens reinforced with recycled PET fibers (*i.e.*, KHSP and CHSP) exhibited significantly larger deflections than the deflections of the Hwangtoh concrete specimens without recycled PET fibers (*i.e*., KH, CH, KHS, and CHS). These findings validate the addition of recycled PET fibers in Hwangtoh concrete to improve the crack-controlling capacity of RC members.

## 5. Conclusions

This study evaluates the material, durability, and structural capacities of Hwangtoh concrete. Plain and RC specimens cast with Korean and Chinese Hwangtoh admixtures and short recycled PET fibers are considered herein. The results of basic material property tests and RC structural member capacity tests are summarized as follows.

(1) From the trial tests, the optimum replacement ratio of Hwangtoh powder to cement is 20%. In addition, without using a chemical water-reducing admixture, desired workability can be achieved through the addition of blast furnace slag powder. By replacing cement with Hwangtoh and blast furnace slag powders in proportions of up to 50%, eco-friendly Hwangtoh concrete with material properties equivalent to the properties of OPC concrete can be developed.
(2) According to the results of the tests herein, Hwangtoh concrete exhibits a slightly lower compressive strength and elastic modulus than plain concrete. However, the differences (less than 2%–6%) are minute, thus, validating the possibility of using Hwangtoh concrete as a structural construction material.
(3) The free drying shrinkage strain of Hwangtoh concrete is approximately 1.5 to 2.0 times greater than the free drying shrinkage strain of plain concrete. The cracking time of Hwangtoh concrete is approximately five days earlier than the cracking time of plain concrete. However, the cracking time of the recycled PET fiber-reinforced Hwangtoh concrete is similar to the cracking time of plain concrete. Accordingly, these finding suggest that the crack-prone characteristics of Hwangtoh concrete can be mitigated by adding short plastic fibers, such as PP or PET fibers, to the material.
(4) Hwangtoh concrete measures slightly lower pH values than the pH values of ordinary cement concrete. The carbonation depth of Hwangtoh concrete is larger than the carbonation depth of ordinary cement concrete due to the decreased production of calcium oxide caused by the replacement of ordinary cement with Hwangtoh. Creep strain is highest in Hwangtoh-PET fiber concrete, followed by slag cement concrete and Hwangtoh concrete. These findings show that there is a tradeoff between drying shrinkage strain resistance and creep strain resistance, which comes with the addition of PET fibers.
(5) The flexural failure behavior of Hwangtoh concrete RC beams is very similar to the flexural failure behavior control specimens herein. The fiber-bridging capability of members reinforced with recycled PET fibers in the tensile region improves the flexural capacity of the RC members by delaying ultimate failure.
(6) This study’s evaluation of the ductility index reveals almost no difference between Hwangtoh and plain concrete without recycled PET fibers. RC specimens with recycled PET fibers, however, have relative ductility indices that are approximately twice as great as the ductility indices of concrete without recycled PET fibers. Therefore, the ductility of RC members cast with Hwangtoh concrete is significantly improved by the addition of recycled PET fibers.
(7) Since all of the performance evaluations are conducted under normal environments in dry condition, additional durability tests of the material in a variety of aggressive environments, such as seawater, must be addressed in future works.
